# Red-backed Shrikes (*Lanius collurio*) resist acoustic mimicry by the Common Cuckoo (*Cuculus canorus*)

**DOI:** 10.1007/s10071-025-02029-x

**Published:** 2025-12-02

**Authors:** Agnieszka Sulej, Iris Charalambidou, Artur Golawski

**Affiliations:** 1 Faculty of Sciences, University of Siedlce, Prusa 14, 08-110 Siedlce, Poland; 2 Department of Life Sciences School of Life and Health Sciences, University of Nicosia, Nicosia, Cyprus

**Keywords:** Acoustic perception, Behavioural flexibility, Brood parasitism, Cuculus canorus, Lanius collurio, Vocal mimicry

## Abstract

The vocal mimicry exhibited by female Common Cuckoos (*Cuculus canorus*), specifically their imitation of raptor calls, has been proposed as a strategy to reduce aggression from host species. Although this hypothesis has been challenged by studies using visual models of cuckoos and hawks, playback-only experiments support the idea of vocal mimicry, i.e. that cuckoo (brood parasite) calls may acoustically mimic those of predators. In this study, we tested the responses of the Red-backed Shrike (*Lanius collurio*), a known host species, to three types of acoustic stimuli: the female cuckoo’s “bubbling call” (which mimics a predator), the call of the Eurasian Sparrowhawk (*Accipiter nisus*), and a control call from the Eurasian Collared Dove (*Streptopelia decaocto*). Playback experiments were conducted both on the breeding grounds in Poland and during migration in Cyprus. Our results revealed that while both cuckoo and sparrowhawk calls elicited behavioural responses, these reactions were clearly distinct. Shrikes responded to sparrowhawk calls by hiding, indicative of predator avoidance, whereas cuckoo calls triggered alertness or approach behaviours. Notably, no response to cuckoo calls was observed during migration, outside the breeding context. This seasonal variation, along with the limited defensive response to cuckoo calls, indicates that vocal mimicry in this context may not serve a protective function. Instead, our findings highlight the shrikes’ nuanced ability to discriminate between acoustic stimuli and respond adaptively based on ecological context. These findings challenge the broad applicability of the predator mimicry hypothesis in cuckoos and emphasise the need for further comparative studies involving diverse host species and multimodal (audiovisual) stimuli.

**Supplementary Information:**

The online version contains supplementary material available at 10.1007/s10071-025-02029-x.

## Introduction

Brood parasites represent approximately 1% of all bird species (Payne 1977) and have evolved a diverse array of strategies to exploit the parental care of other species (Davies 2000). The significant fitness costs imposed by brood parasitism exert strong selective pressure on host species, driving the evolution of a wide range of defensive adaptations (Davies 2000; Trnka and Grim 2014; Soler 2017; Yang et al. 2022). In turn, brood parasites have developed counter-adaptations to overcome these defenses, resulting in a classic coevolutionary arms race between parasite and host (Davies 2000; Feeney et al. 2012). The first line of defence against brood parasitism often involves strategic nest site selection, with hosts avoiding areas frequently visited by parasites (Liang et al. 2013; Tryjanowski et al. 2024). Beyond spatial avoidance, hosts employ a range of active defensive behaviours in response to the presence of a parasite, such as mobbing and direct physical attacks (Welbergen and Davies 2009; Moksnes et al. 2013; Campobello et al. 2017), which in extreme cases may lead to the intruder’s death (Šulc et al. 2020). In addition to overt physical aggression, hosts often display less intense defensive behaviour, such as alarm calling (Thys et al. 2019; Tryjanowski et al. 2025), tail-flicking as a display of agitation (Polak 2019), or cautious approaches towards the intruder to assess or deter the threat (Davies and Welbergen 2009). In response to host defensive behaviours, brood parasites have evolved mimetic traits resembling species perceived as dangerous by hosts. One of the most well-documented examples in birds is the visual and vocal mimicry exhibited by the female Common Cuckoo (*Cuculus canorus*; hereafter “cuckoo”) toward the Eurasian Sparrowhawk (*Accipiter nisus*). Visual mimicry includes characteristics such as a slender body shape, grey plumage, ventral barring, yellow eyes and legs, and a hawk-like flight pattern – traits that closely resemble those of the sparrowhawk (Welbergen and Davies 2011; Go et al. 2021). In addition to visual resemblance, female cuckoos produce “bubbling” calls that acoustically mimic sparrowhawk vocalizations (York and Davies 2017; Wang et al. 2021). This dual mimicry strategy is thought to deter hosts by triggering anti-predator responses, into avoiding the source of the sound (i.e., the vicinity of the nest), thereby increasing the likelihood that the cuckoo will lay its egg undetected (Marton et al. 2021).

Numerous experimental studies have investigated host responses to both cuckoo and sparrowhawk models (Davies and Welbergen 2008; Ma et al. 2018; Krausová et al. 2022). These investigations consistently demonstrate that hosts often struggle to differentiate between the two, exhibiting similar behavioural reactions to sparrowhawk and grey morph cuckoo dummies (Davies and Welbergen 2008; Møller et al. 2015; Ma et al. 2018). These findings are further supported by spontaneous field observations (Lyon and Gilbert 2013), and experimental evidence confirms the reliability of using simulated stimuli in cuckoo brood parasitism studies, as hosts respond in comparable ways to models and live cuckoos (Tryjanowski et al. 2018). Nevertheless, certain species, such as members of the *Acrocephalus* genus and the Barn Swallow (*Hirundo rustica*), have demonstrated the ability to distinguish between these threats (Duckworth 1991; Trnka and Grim 2013; Liang and Møller 2015).

The role of vocal cues in host-parasite interactions, particularly in the absence of visual stimuli, has received comparatively less attention. However, existing studies suggest that the bubbling calls of female cuckoos may be acoustically similar to those of sparrowhawks, leading to potential misidentification by host species. For example, Zhang et al. (2021) reported that four bird species, including two cuckoo hosts, exhibited comparable responses to the calls of female cuckoos and sparrowhawks. Similar findings have been documented in the Eurasian Reed Warbler (*Acrocephalus scirpaceus*) (York and Davies 2017) and the Oriental Reed Warbler (*A. orientalis*) (Wang et al. 2022). Moreover, female cuckoo calls elicited similar levels of attentiveness as sparrowhawk calls in overwintering Great Tits (*Parus major*) and Blue Tits (*Cyanistes caeruleus*) - two species rarely targeted by cuckoos (Grim et al. 2014). Interestingly, comparable responses have also been observed in domestic chickens (*Gallus gallus domesticus*), suggesting a high degree of vocal mimicry between the two species (Jiang et al. 2021). To date, there is no conclusive evidence that host species can reliably distinguish the call of the female cuckoo from that of the sparrowhawk in the absence of visual cues. This underscores a significant gap in our understanding of the role of vocal mimicry in host–parasite interactions.

Consequently, a key question arises: can hosts recognise the brood parasite solely on acoustic signals, or is visual confirmation essential for accurate threat assessment? Given the cuckoo’s secretive egg-laying behaviour (Thorogood and Davies 2016), vocal mimicry may serve as an additional strategy to enhance parasitic success by reducing host vigilance. Addressing this question is crucial for advancing our understanding of the coevolutionary dynamics between brood parasites and their hosts, and for refining theoretical models of evolutionary arms races. The Red-backed Shrike (*Lanius collurio*) has historically been a widespread European host of the cuckoo. Although subject to frequent parasitism in the past (Münster 1958; Gotzman and Jabłoński 1972), current parasitism rates have declined to below 1% in most populations (Adamík et al. 2009; Soler et al. 2012; Wesołowski and Mokwa 2013). For these reasons, the species can be regarded as a recently abandoned host of the cuckoo, in which the co-evolutionary arms race has already been terminated (sensu Dawkins and Krebs 1979). This decline is likely attributable to the species’ improved ability to recognise and reject foreign eggs (Lovászi and Moskát 2004; Krausová 2023). Red-backed Shrikes are capable of distinguishing cuckoos from sparrowhawks based on visual cues from dummy models (Krausová et al. 2022), and their combination of high aggression and advanced cognitive abilities facilitate effective threat detection (Strnad et al. 2012; Polak 2019). These traits may have contributed to the reduced success of cuckoo parasitism in this host species (Krausová et al. 2022). Since anti-parasitism behaviours may persist for many years after the cessation of parasitism (Rothstein 2001; Hale and Briskie 2007), the Red-backed Shrike represents an excellent model for studying parasite recognition, offering a unique combination of historical exposure to parasitism, strong defensive behaviour, and notable cognitive flexibility. The present study aimed to evaluate whether Red-backed Shrikes can distinguish between the calls of female cuckoos and sparrowhawks in the absence of visual cues. Experiments were conducted both on the breeding and migratory grounds of this species. In contrast to previous studies that primarily emphasised aggressive responses, our approach also incorporated behaviours associated with threat avoidance, allowing for a more nuanced analysis of the birds’ reactions. Additionally, we explored the potential for context-dependent variation in responses between breeding and migration periods, offering a more comprehensive understanding of the mechanisms underlying threat perception.

The experiment involved playback of female cuckoo and sparrowhawk calls in two distinct ecological contexts: (1) within breeding territories, where cuckoos pose a genuine threat to reproductive success through brood parasitism; and (2) at migratory stopover sites, where this threat is absent. Two competing hypotheses were tested: (1) If vocal mimicry by cuckoos is effective, shrikes should respond similarly to both calls in both contexts by avoiding the sound source; (2) Alternatively, if shrikes can discriminate between the calls, they should exhibit heightened aggression towards the cuckoo call during the breeding season - perceiving it as a parasitic threat - while during migration, they should avoid only the sparrowhawk call and disregard the cuckoo call as irrelevant. Notably, the geographic ranges of the Red-backed Shrike, the cuckoo, and the sparrowhawk largely overlap during both breeding and migratory periods (BirdLife International 2025), increasing the likelihood of frequent encounters and maintaining selective pressure for accurate threat discrimination.

## Materials and methods

### Study species

The Red-backed Shrike is a small, territorial passerine with a broad distribution across Europe and western Asia, though its population is currently in decline (BirdLife International 2025). This species undertakes long-distance migrations, typically arriving at its breeding grounds from Africa in April/May (Lefranc 2022). In eastern Poland, the majority of the population occupies agricultural landscapes, nesting along woodland edges, in tree clumps, orchards, and currant plantations (Golawski and Meissner 2008). The breeding season generally begins in mid-May and continues through August. Clutches usually consist of three to seven eggs, which are incubated for approximately 14–15 days. Nestlings remain in the nest for about 15 days, and fledglings stay near the nest for another 2–4 weeks (Lefranc 2022). Although typically single-brooded, the species frequently lays replacement clutches following first-brood failure (Antczak et al. 2009). The Red-backed Shrike is primarily insectivorous (Tryjanowski et al. 2003; Morelli et al. 2015). Both the cuckoo and the Eurasian Sparrowhawk are widespread breeding species in the region and are commonly encountered during the shrike’s breeding season (Tomiałojć and Stawarczyk 2003).

### General protocol

Behavioural responses were assessed in both breeding pairs and solitary individuals. Subjects were exposed to three-minute playback trials featuring one of three vocalization types: female cuckoo calls, Eurasian Sparrowhawk calls, or male Eurasian Collared Dove (hereafter “dove”) vocalizations, serving as a control. Each playback stimulus was prepared from a single recording obtained from the Xeno-Canto database (see: Blackburn et al. 2014). From each recording, we extracted one clear sequence of the target species’ call, free of background noise or overlapping sounds. This sequence was then repeated six times within a 3-min playback file, separated by silent intervals of equal length, so that each playback consisted of six call sequences evenly spaced across the trial (see details: Table S1). To avoid pseudoreplication, each pair or individual was tested only once with a single playback type. To further reduce pseudoreplication, three distinct recordings per playback type were randomly assigned across trials (Kroodsma et al. 2001). Audio playback was delivered using Bluetooth wireless speakers (JBL 5) connected via smartphone, with sound amplitude standardised across all trials to reflect natural vocalisation levels (80–95 dB SPL at 1 m; Xia et al. 2019). To reduce observer-induced startle responses, playback was initiated 2 min after the speaker had been placed, allowing the birds to settle before the experiment began (Cunningham and Magrath 2017). A single, randomly selected playback type was presented near a nest site or a migrating shrike. Bird behaviour was monitored over a five-minute observation period, comprising three minutes during playback and two minutes post-playback, by a concealed observer positioned approximately 30 m from the speaker. Behaviour was classified as ‘natural’ (i.e., non-reactive) if individuals continued baseline activities without noticeable deviation in response to the heterospecific playback. These baseline behaviours included perching, foraging, short-distance movements for perch changes or prey delivery, and calm resting. In contrast, reactive behaviours were defined as any departure from baseline activity during or immediately following playback, such as: direct orientation and approach towards the speaker, tail wagging, alarm calling, vigilant scanning, upward glancing, freezing, or retreating into cover (Caro 2005).

### Breeding season

Field experiments were conducted in eastern Poland (52.04–52.35° N, 21.86–22.44° E) between May and July of 2023 and 2024. Shrikes were observed nesting in a variety of habitats, including fallow lands, roadside vegetation strips, woodland edges, mid-field tree clumps, and riparian shrub belts. Clutch age was determined prior to experimentation using the water test method (Wesołowski 1986), a method recognised for its accuracy and safety for developing embryos (Liebezeit et al. 2007). Two eggs per clutch (separately) were immersed in water-filled containers, and their position was used to estimate incubation stages. This approach allows relatively precise ageing up to the fifth day of incubation, based on characteristic changes in egg position. Playback stimuli were delivered via a speaker positioned 5 m from the nest, allowing for unobstructed observation of parental behaviour from a concealed distance (Lawson et al. 2021; Tryjanowski et al. 2021).

Initial behavioural responses were recorded on a binary scale: 0 – no response, 1 – response. To capture more nuanced variation, we also applied a four-point ordinal scale: (A) No response, (B) Concealment within vegetation during playback, (C) Distant reaction without approaching the speaker, (D) Intense response involving direct approach toward the speaker. Concealment (B) was recorded when individuals withdrew directly into dense vegetation during playback and remained hidden for its duration. Category (C) compromised all signs of distant agitation without approaching the speaker, such as tail wagging, calling, nervous looking around, looking at the sky, and freezing. Approaching the speaker (D) was defined as any distinct displacement in its direction, typically exceeding 1 m, combined with sustained orientation towards the playback source. Because parental activity in the vicinity of nests varied between trials, only clearly visible adults were considered in the analyses. This classification follows Polak (2019) and Wang and Yang (2020), with the addition of category (B) to account for concealment behaviour, which has been documented in host species exposed to predator cues (Welbergen and Davies 2008). In total, the playback experiment included 25 trials with cuckoo calls, 22 with sparrowhawk calls, and 15 with collared dove calls, with each nest tested only once.

### Migration period

Experiments conducted during the autumn migration period were carried out in southeastern Cyprus (34.97–35.00° N, 34.04–34.07° E), a region outside the breeding range of the species. The study was scheduled to align with the peak migratory passage of Red-backed Shrikes in September 2023 (Roth 2008). Observations were made in naturally regenerating habitats characterised by sparse, scattered shrubs and trees, where individuals frequently perched to scan for prey. The dominant vegetation in the area was Phoenician juniper (*Juniperus phoenicea*).

A loudspeaker was placed no more than 30 m (mean = 26.5 m, Range 23–29 m) from the observed shrike. Behavioural responses were recorded using a binary scoring system: 0 indicating no response, and 1 indicating a response. A response was scored whenever the focal individual displayed any deviation from calm baseline activity, including vigilance or withdrawal into vegetation, corresponding to categories C or B in the breeding context. In contrast to the breeding season, no approaches toward the speaker (category D) were observed during migration. Each experiment targeted a single, clearly separated individual, and playback was initiated only once the focal bird had been observed in a calm state, showing baseline behaviour and no reaction to the observer. Although experiments were conducted within the same general area, only newly encountered individuals were tested each day. The number and composition of shrikes varied markedly between consecutive days, suggesting high turnover and short stopover durations. Thus, while pseudoreplication cannot be entirely excluded due to the absence of individual marking, the likelihood of testing the same bird more than once was considered low. The vocal playback experiment comprised 47 cuckoo calls, 48 Eurasian Sparrowhawk calls, and 27 Eurasian Collared Dove calls.

### Statistical analyses

All statistical analyses were performed using R version 4.3.3 (R Core Team 2024). Separate generalised linear models (GLMs) were constructed for the breeding and migratory contexts to identify predictors of behavioural responses. Sex was excluded as a covariate, as previous studies have found no sex-related differences in this species’ response to brood parasites or predators (Tryjanowski and Goławski 2004; Polak 2019). Our own statistical comparisons confirmed these findings: the occurrence of a response versus no response did not differ among males, females, and pairs (where both members showed the same outcome) (chi² = 4.05, df = 2, *p* = 0.132; Table S2), and the distribution of the four response categories (A–D) was likewise similar between sexes (chi² = 1.43, df = 3, *p* = 0.699; Table S3). These results support the conclusion that pooling sexes in the main analyses is justified. Interaction terms were omitted due to their lack of statistical significance.

In the breeding season model, the following explanatory variables were included: (1) time – time of day, categorised as 08:01–14:00 vs. 14:01–20:00, to reflect diel variation in cuckoo activity (Davies 2000), (2) stage – breeding stage, classified to represent varying vulnerability to parasitism, with stage 1 spanning from clutch initiation to day 4 of incubation, and stage 2 from day 5 to the end of incubation. This classification reflects critical periods of vulnerability identified in cuckoo–host studies (Wang et al. 2022). (3) laying egg, recorded as the Julian date of the first egg laid (May 1= day 1) (Brooke et al. 1998; Wang and Yang 2020), and (4) playback species, comprising female cuckoo, sparrowhawk, and collared dove, with the latter serving as a control species due to its lack of threat relevance. The response variable was binary (0 = no response, 1 = response). A binomial GLM identified the model including laying egg and playback species as the best fit (see Table 1), based on AIC values. Model selection followed an information-theoretic approach, with models within ΔAIC ≤ 2 considered equally supported (Burnham and Anderson 2002). Predicted response probabilities were visualised using the ggpredict() function from the ggeffects package (Lüdecke 2018). Additionally, the distribution of detailed response types (A–D) across playback categories was compared using Fisher’s exact test (fisher.test() in R). Post hoc pairwise comparisons were performed using Tukey’s Honestly Significant Difference (HSD) test, based on estimated marginal means derived from a GLM (emmeans package). In the migration model, the predictors included: (1) birds were classified as juveniles (first-year individuals in characteristic spotted plumage) or adults (birds in adult male- or female-type plumage), to test for possible ontogenetic effects on cue recognition, (2) species – playback type, comprising female cuckoo, sparrowhawk, or collared dove calls. The dependent variable was binary (1 = response, 0 = no response). Based on AIC values, the best-fitting model retained only species as a significant predictor (Table 1). Response probabilities were estimated using the ggpredict(), and post hoc pairwise comparisons were conducted using Tukey’s HSD test on estimated marginal means derived from the corresponding GLM.

**Table 1:** Ranking of candidate GLM models evaluating predictors of Red-backed shrike response probability to playback calls during the breeding and migration periods. Variables include playback species, clutch initiation date, breeding stage, time of day, and bird age. Abbreviations: df = degrees of freedom; LL = log-likelihood; AICc = corrected Akaike information Criterion; ΔAICc = difference from top-ranked model; AICcwt = model weight

Period	Model parameters	df	LL	AICc	Δ AICc	AICwt
Breeding period	Laying date + Species	4	−42.46	93.6	0.00	0.386
	Laying date + Species + Time	5	−42.22	95.5	1.89	0.151
	Laying date + Species + Stage	5	−42.40	95.9	2.24	0.126
	Species	3	−44.73	95.9	2.26	0.125
Migration	Species	3	−85.80	177.8	0.00	0.706
	Age + Species	4	−85.61	179.6	1.75	0.294

## Results

### Breeding season

**Fig. 1:**
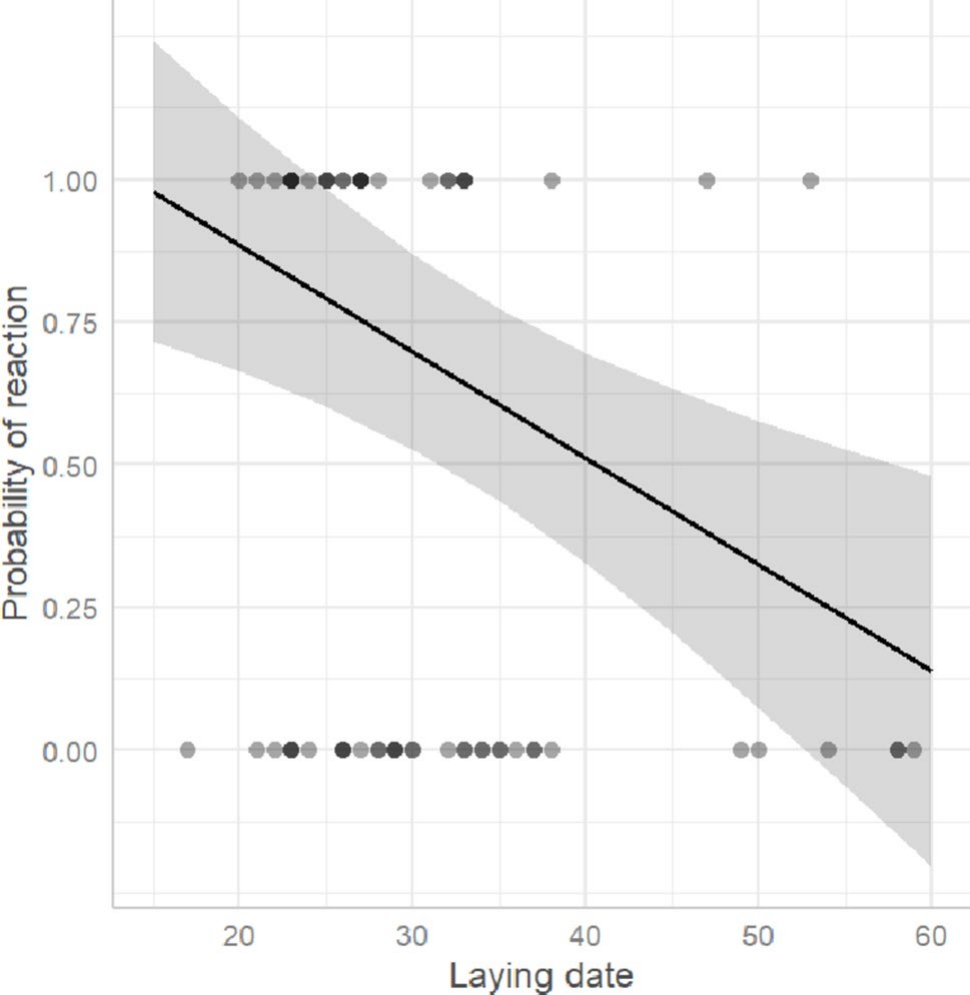
Predicted probability of Red-backed Shrike responses during the breeding season in relation to clutch initiation date. The regression line and 95% confidence intervals are derived from the best-fitting GLM. Each dot represents an observation; the intensity of shading indicates the density of overlapping observations at each time point, representing either absence (0) or presence (1) of a reaction

**Fig. 2:**
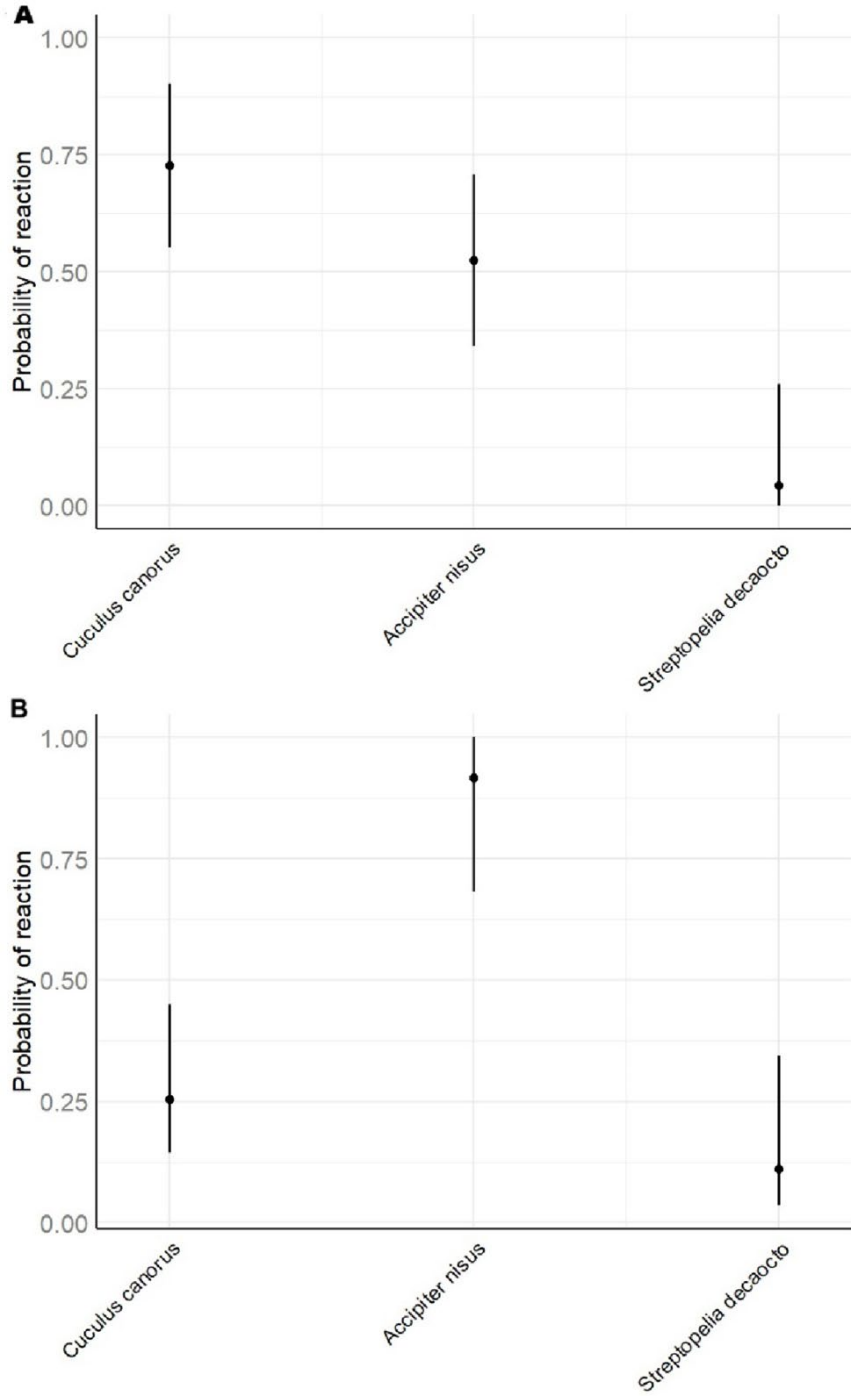
Model-predicted probabilities of Red-backed Shrike responses to different playback stimuli during the breeding season (panel A) and the migration period (panel B). Points represent predicted means; vertical lines indicate 95% confidence intervals

**Fig. 3:**
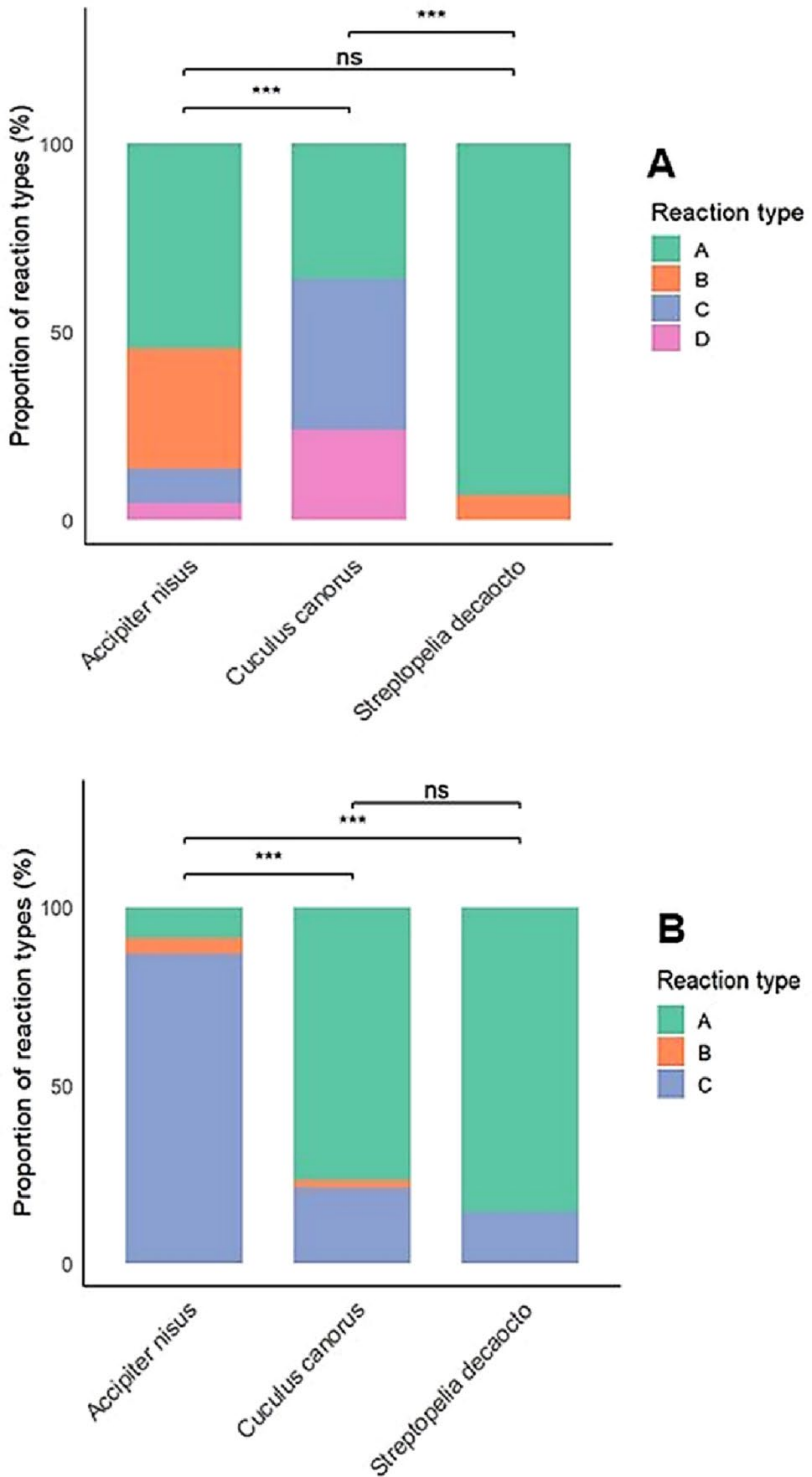
Distribution of behavioural response types exhibited by Red-backed Shrikes in reaction to different playback calls during the breeding season (panel A) and the migration period (panel B). Response categories: (A) no reaction; (B) hiding in vegetation; (C) distant response without clear approach; (D) strong approach toward the speaker. Statistical significance: ns = p >0.05; *** = p ≤ 0.001

**Fig. 4:**
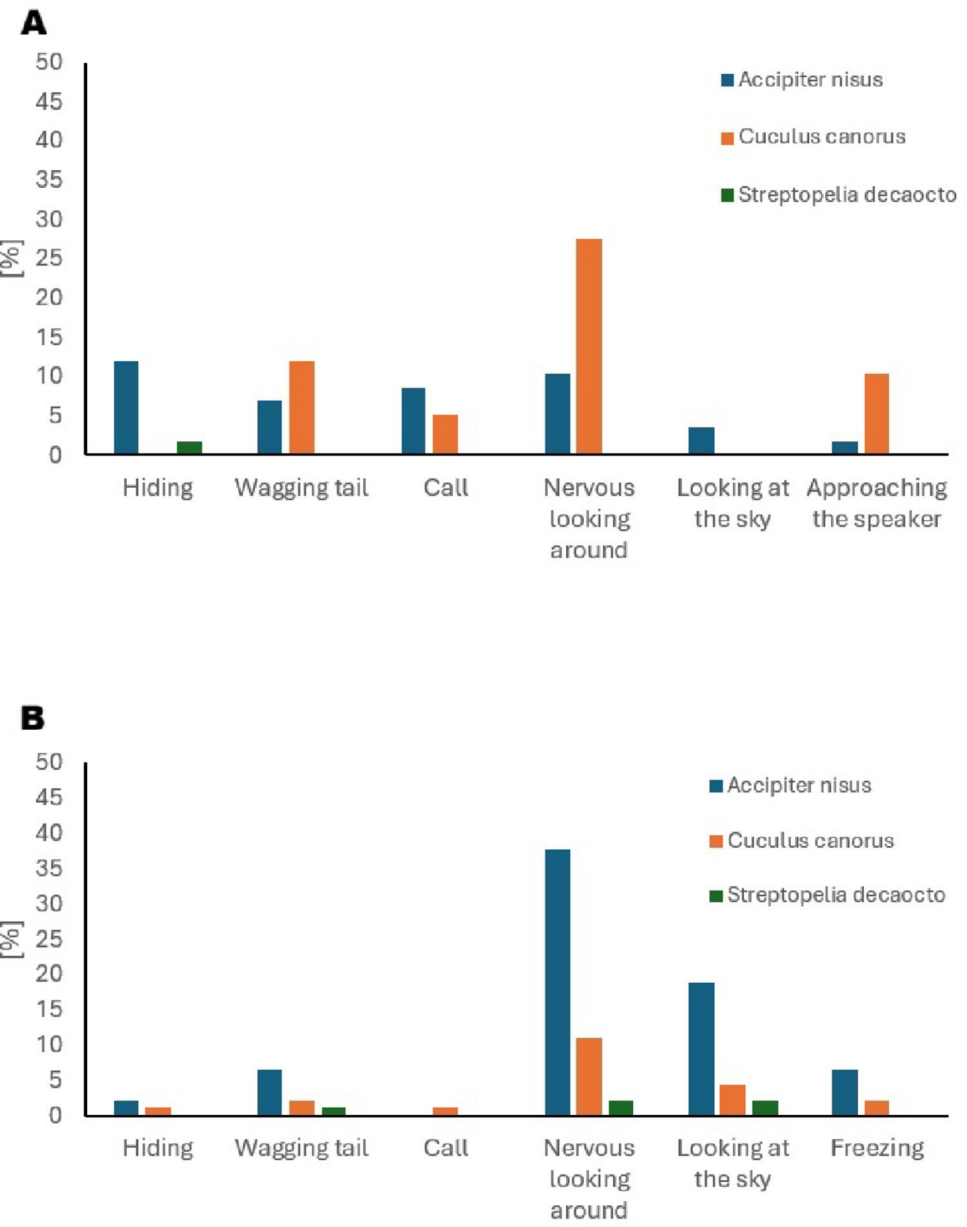
Breakdown of Red-backed Shrike behavioural responses by type and playback stimuli during the breeding season (panel A) and the migration period (panel B)

Experiments were conducted on 62 nests during the egg-laying and incubation stages. The GLM model revealed that both laying date (GLM, *p* < 0.01) and playback type (*C. canorus*: GLM, *p* < 0.001; *A. nisus*: GLM, *p* < 0.01) significantly predicted the overall likelihood of a behavioural response (Table 2). Shrikes showed a higher probability of responding earlier in the season; for example, when the first egg was laid on 15 May, the predicted probability of response was 98.0%, whereas for clutches initiated on 29 June, it dropped to 14.0% (Fig. 1). Average response probabilities by playback type were 73.0% for cuckoo, 52.0% for sparrowhawk, and only 0.04% for the control species, the Eurasian collared dove (Fig. 2A). Tukey’s HSD confirmed that responses to both cuckoo and sparrowhawk calls were significantly more frequent than to dove calls (*C. canorus* vs. *S. decaocto*: *p* < 0.001; *A. nisus* vs. *S. decaocto*: *p* = 0.004), while the difference between cuckoo and sparrowhawk responses was not significant (*p* = 0.231) (Table 3). Although the binary comparison showed no difference between cuckoo and sparrowhawk treatments, analysis of response types (A–D) revealed significant differences in their structure (Fisher’s exact test, *p* < 0.001). Cuckoo calls more often elicited intense approach behaviour, whereas sparrowhawk calls more frequently triggered concealment in vegetation (Fig. 3A). Within the behaviours classified as category C, shrikes notably exhibited looking around more frequently in response to female cuckoo calls than to the other two playbacks, whereas looking up at the sky occurred exclusively in response to sparrowhawk calls (Fig. 4A).

**Table 2:** Estimated coefficients from the best-performing GLMs explaining variation in Red-backed shrike response probability during the breeding and migration periods. Significant predictors include playback species and clutch initiation date

Period	Parameters	Estimate	SE	t-test	*p*
Breeding period	Intercept	0.576	0.186	3.09	< 0.01
	Laying date	−0.019	0.006	−3.36	< 0.01
	*Accipiter nisus*	0.480	0.143	3.36	< 0.01
	*Cuculus canorus*	0.683	0.141	4.86	< 0.001
Migration	Intercept	−2.197	0.577	−3.81	< 0.001
	*Accipiter nisus*	2.110	0.597	3.54	< 0.001
	*Cuculus canorus*	0.832	0.646	1.29	0.197

**Table 3:** Tukey’s HSD post hoc comparisons of Red-backed shrike response frequencies among playback stimuli. Significant differences are indicated by adjusted p-values

Period	Pairwise comparison	Estimate	SE	df	t-test	*p*
Breeding period	*Streptopelia decaocto* – *Accipiter nisus*	−0.480	0.143	58	−3.36	0.004
	*Streptopelia decaocto* – *Cuculus canorus*	−0.683	0.141	58	−4.86	< 0.001
	*Accipiter nisus* – *Cuculus canorus*	−0.203	0.123	58	−1.66	0.231
Migration	*Streptopelia decaocto* – *Accipiter nisus*	−0.806	0.086	119	−9.35	< 0.001
	*Streptopelia decaocto* – *Cuculus canorus*	−0.144	0.087	119	−1.67	0.222
	*Accipiter nisus* – *Cuculus canorus*	0.661	0.074	119	8.99	< 0.001

### Migration period

During the migration period, behavioural experiments were conducted on 122 individual shrikes. The GLM showed that playback type was the only significant predictor of response. Specifically, the sparrowhawk call elicited a strong effect (*A. nisus*: GLM, *p* < 0.001), while the cuckoo call did not differ significantly from the control (GLM, *p* = 0.197) (Table 2). The sparrowhawk call elicited the highest response rate, with 92.0% of individuals reacting, compared with 26.0% for the cuckoo and 11.0% for the dove (Fig. 2B). Tukey’s HSD test confirmed that responses to the sparrowhawk were significantly greater than to both the cuckoo and the dove (*p* < 0.001 for both comparisons), while the difference between cuckoo and dove responses was not statistically significant (*p* = 0.222) (Table 3). During this period, shrikes markedly and consistently exhibited the full spectrum of behaviours assigned to category C in response to sparrowhawk calls, significantly more often than in response to the other two species (Figs. 3B and 4B).

## Discussion

Our experiments during the breeding season revealed clear differences in shrike responses to sparrowhawk and cuckoo calls. Contrary to the suggestions that the female cuckoo’s “bubbling” call functions as effective predator mimicry to deter host aggression (York and Davies 2017; Jiang et al. 2021; Marton et al. 2021), our findings do not support this hypothesis. During the breeding season, sparrowhawk calls consistently triggered avoidance behaviours - primarily hiding - while the cuckoo call elicited exploratory or ambiguous anxiety-like responses. The Red-backed Shrike’s ability to discriminate between acoustically similar calls indicates that the female cuckoo’s vocalization does not serve as effective predator mimicry in this species. The absence of response to the control stimulus - the collared dove - further supports the ecological specificity and relevance of shrike reactions. These results are consistent with previous studies using visual decoys, which also reported limited effectiveness of cuckoo mimicry in this shrike (Krausová et al. 2022). In natural settings, shrikes typically avoid sparrowhawks by retreating from the nest and refraining from aggressive defence (Welbergen and Davies 2008; Trnka and Prokop 2012; Ma et al. 2018; Krausová et al. 2022), reinforcing the interpretation that predator avoidance, rather than mimicry-driven deception, underlies their behavioural responses. In addition, field observations indicated that both members of a pair often attended the nest area during an experiment, yet we did not observe consistent differences between sexes in the likelihood of responding. This supports the conclusion that male and female shrikes contribute similarly to nest defence and can be analysed jointly at the pair level.

Analyses of specific behavioural responses revealed qualitative differences between shrike reactions to cuckoo and sparrowhawk calls. While previous research has largely focused on response rates, often overlooking the diversity of behavioural outcomes (Polak 2019; Wang and Yang 2020) our findings emphasise the importance of response type in interpreting host perception. If the female cuckoo’s call functioned as effective predator mimicry, one would expect consistent avoidance behaviours, such as retreat or hiding, - which were not observed. Instead, shrike responses were more exploratory or ambiguous, suggesting that the cuckoo call is interpreted as a non-specific cue rather than a clear signal of danger. Although the predator-mimicry hypothesis remains the prevailing explanation for the female cuckoo’s call, growing evidence indicates that this vocalization may serve multiple functions. It could play diverse roles in both intra- and interspecific interactions, including territorial signaling among females and mate attraction (Moskát and Hauber 2019, 2023; Xia et al. 2019; Hauber and Moskat 2025; Jin et al. 2025). Our findings suggest that the absence of strong defensive responses in shrikes may not indicate a failure of mimicry, but rather reflect the limited relevance of this signal within the specific context of the cuckoo-host relationship.

During migration, shrikes responded strongly to sparrowhawk calls but largely ignored cuckoo calls. This seasonal contrast suggests that the cuckoo call is not perceived as a general threat signal. Such seasonal shifts in behaviour align with previous findings on avian cognitive flexibility and the contextual evaluation of risk (Thorogood and Davies 2016; Soler 2017). Defensive responses appear to be modulated by ecological factors such as breeding stage, presence of offspring, and territorial status. For instance, reed warblers adjust their responses to cuckoos based on a combination of personal experience and social cues (Thorogood and Davies 2016), while host tolerance outside the breeding season may represent an adaptive (Grim 2007), energy-conserving strategy within the broader risk-cost trade-off of anti-parasitic defence behaviour.

The absence of response to cuckoo calls during migration, contrasted with consistent reactions to sparrowhawk calls, highlights shrikes’ ability to discriminate between stimuli and adjust behaviour seasonally. According to Soler (2017), such cognitive flexibility is the result of long-term coevolution, enabling hosts to assess threats in an adaptive and context-dependent manner.

Shrike responses declined later in the breeding season, with stronger reactions observed at earlier clutches. This may reflect variation in female condition and experience, as early breeders are often of higher individual quality, which can influence their investment in costly defensive behaviours (Sæther 1990; Ardia and Clotfelter 2007). In contrast, younger or less experienced females may be less inclined to engage in energetically demanding responses (Hamao 2011). Additionally, replacement clutches following earlier reproductive failures may involve reduced parental investment and limited resource availability, further diminishing the likelihood of strong defensive reactions (Antczak et al. 2009; Redmond et al. 2020). As with many field studies, our research is subject to certain limitations. The experiments were conducted in two geographically distinct regions - eastern Poland and southeastern Cyprus - which may have influenced host behaviour due to regional differences in exposure to cuckoos. Moreover, breeding-season trials were spread across two years, introducing the possibility of retesting the same individuals. However, given the low philopatry observed in shrikes in Poland (Tryjanowski et al. 2007), the likelihood of repeated sampling is minimal and unlikely to have significantly affected the results.

The results of this study provide important insights into acoustic signal perception in the Red-backed Shrike, a known host of the cuckoo. The experiments demonstrated that both the female cuckoo’s characteristic “bubbling” call and the sparrowhawk call elicited behavioural responses, but the nature of these responses differed markedly. This distinction has important implications for understanding the effectiveness of vocal mimicry and provides the first evidence that hosts may discriminate between threats based solely on acoustic cues, without requiring visual confirmation of a brood parasite or predator. By analysing shrike responses to cuckoo and sparrowhawk calls across two contrasting ecological contexts, namely breeding territories and migration stopovers, the study deepens our understanding of the functional role of vocal mimicry in natural settings. The results highlight the context-dependent nature of threat recognition and underscore the role of acoustic cues in the evolutionary arms race between brood parasites and their hosts.

In summary, our findings challenge the prevailing hypothesis that the female cuckoo’s bubbling call functions as effective acoustic predator mimicry. Instead, shrikes appear capable of discriminating the call as non-threatening, with responses shaped by both behavioural cues and seasonal context. Defensive behaviour was observed during the breeding season but was absent during migration, reflecting the species’ high cognitive plasticity and advanced adaptive capabilities. These results support the view of a dynamic evolutionary arms race in which the parasite’s strategies are increasingly constrained by the host’s cognitive abilities. Future studies should include other host species with varying degrees of coevolutionary history with cuckoos. Particularly promising are experimental designs that integrate both acoustic and visual stimuli, offering a more comprehensive understanding of the functional role of the cuckoo’s complex signaling repertoire.

## Supplementary Information

Below is the link to the electronic supplementary material.

Supplementary Material 1

## Data Availability

No datasets were generated or analysed during the current study.
