# Supplementary material for: Red-backed Shrikes (Lanius collurio) resist acoustic mimicry by the Common Cuckoo (Cuculus canorus)
Source: Anim Cogn. 2025 Dec 2;29(1):9. doi: 10.1007/s10071-025-02029-x (PMC12799621; doi:10.1007/s10071-025-02029-x)
Supplement: Supplementary file 1 — Supplementary Material 1 [file 10071_2025_2029_MOESM1_ESM.docx]

Supplementary materials

Red-backed Shrikes (*Lanius collurio*) resist acoustic mimicry by the Common Cuckoo (*Cuculus canorus*)

Agnieszka Sulej^1^*, Iris Charalambidou^2^, Artur Golawski^1^

^1^University of Siedlce, Faculty of Sciences, Prusa 14, 08-110 Siedlce, Poland

^2^University of Nicosia, Department of Life Sciences, School of Life and Health Sciences, Nicosia, Cyprus; charalambidou.i@unic.ac.cy

*corresponding author; e-mail: ap48@stud.uws.edu.pl

**Table S1.** Details of playback stimuli used in the experiments. For each recording (Xeno-Canto ID), the table lists the duration of a single call sequence and the duration of the silent interval between sequences

| Species | Xeno-Canto codes | Duration of one sequence [s] | Interval between sequences [s] |
| --- | --- | --- | --- |
| Cuckoo 1 | XC430909 | 2.0 | 33.6 |
| Cuckoo 2 | XC916983 | 2.5 | 33.0 |
| Cuckoo 3 | XC917246 | 3.1 | 32.3 |
| Collared Dove 1 | XC541633 | 8.1 | 26.3 |
| Collared Dove 2 | XC669773 | 13.4 | 19.9 |
| Collared Dove 3 | XC728164 | 10.4 | 23.5 |
| Sparrowhawk 1 | XC420205 | 3.3 | 32.0 |
| Sparrowhawk 2 | XC741418 | 1.4 | 34.3 |
| Sparrowhawk 3 | XC744165 | 3.1 | 32.3 |

**Table S2.** Presence or absence of response in male, female, and pair Red-backed Shrikes during playback experiments (χ² = 4.05, df = 2, p = 0.132)

|  | Male only | Female only | Both pair members |
| --- | --- | --- | --- |
| No response | 7 | 7 | 35 |
| Response occurred | 7 | 7 | 13 |

**Table S3.** Response categories (A–D) recorded in male and female Red-backed Shrikes during playback experiments (test chi² = 1.43, df = 3, p = 0.699). Categories explanation: (A) No response, (B) Concealment within vegetation during playback, (C) Distant reaction without approaching the speaker, (D) Intense response involving direct approach toward the speaker

| Response category | Male | Female |
| --- | --- | --- |
| A | 42 | 42 |
| B | 5 | 5 |
| C | 9 | 12 |
| D | 6 | 3 |
